# Niagra Falls Academy of Medicine

**Published:** 1906-04

**Authors:** John A. Rafter


### Niagara Falls Academy of Medicine.

Reported by JOHN A. RAFTER, M. D.

The regular monthly meeting of the Academy of Medicine of Niagara Falls was held at the home of Dr. Frank Guillemont, on Tuesday evening, March 19, 1906, Dr. A. L. Chapin in the chair, and Dr. Wixson, secretary, at his desk.

The members present were: Drs. Scott, Griswold, Potter, Miller, McChesney, C. E. Campbell, McCarthy and Guillemont. The guest of the occasion was Dr. Charles E. Congdon, of Buffalo, who read a paper on "The Surgical Treatment of Gallstones." A number of specimens were shown by Dr. Congdon in explanation of his paper.

Drs. Scott, McCarthy, Potter and others discussed the paper. After the business session was over a collation was served.

These monthly meetings of the Academy of Medicine are delightful affairs both from a professional and social standpoint. At each meeting some prominent member of the profession from Buffalo or elsewhere is invited to be present as the guest, and to present a paper. One cannot but be impressed with the spirit of good-fellowship that prevails among the members of the Academy. Niagara Falls is fortunate in having a large number of able men in the medical profession.

